# Serum milk fat globule-EGF factor 8 (MFG-E8) as a diagnostic and prognostic biomarker in patients with hepatocellular carcinoma

**DOI:** 10.1038/s41598-019-52356-6

**Published:** 2019-10-31

**Authors:** Tomonari Shimagaki, Sachiyo Yoshio, Hironari Kawai, Yuzuru Sakamoto, Hiroyoshi Doi, Michitaka Matsuda, Taizo Mori, Yosuke Osawa, Moto Fukai, Takeshi Yoshida, Yunfei Ma, Tomoyuki Akita, Junko Tanaka, Akinobu Taketomi, Rikinari Hanayama, Tomoharu Yoshizumi, Masaki Mori, Tatsuya Kanto

**Affiliations:** 1 Department of Liver Disease, The Research Center for Hepatitis and Immunology, National Center for Global Health and Medicine, Ichikawa, Japan; 2 Department of Surgery and Science, Graduate School of Medical Sciences, Kyushu University, Fukuoka, Japan; 3 Department of Gastroenterological Surgery I, Hokkaido University Graduate School of Medicine, Sapporo, Japan; 4 Department of Immunology, Kanazawa University Graduate School of Medical Sciences, Kanazawa, Japan; 5 Department of Epidemiology, Infectious Disease Control and Prevention, Graduate School of Biomedical and Health Sciences, Hiroshima University, Hiroshima, Japan

**Keywords:** Hepatocellular carcinoma, Tumour biomarkers, Diagnostic markers

## Abstract

Current serum hepatocellular carcinoma (HCC) biomarkers are insufficient for early diagnosis. We aimed to clarify whether serum MFG-E8 can serve as a diagnostic or prognostic biomarker of HCC. Serum MFG-E8 levels of 282 HCC patients, who underwent primary hepatectomy, were examined by ELISA. We also quantified serum MFG-E8 levels in patients with chronic hepatitis (CH), liver cirrhosis (LC), as well as in healthy volunteers (HVs). Serum MFG-E8 levels were significantly lower in HCC patients than in HVs regardless of the etiology of liver disease (3.6 ± 0.1 vs 5.8 ± 0.2 ng/mL, p < 0.0001), and recovered after treatment of HCC. Serum MFG-E8 levels in CH and LC patients were comparable to those in HVs. Serum MFG-E8 could detect HCCs, even α-fetoprotein (AFP)-negative or des-γ-carboxy prothrombin (DCP)-negative HCCs, in CH and LC patients. Our new HCC prediction model using MFG-E8 and DCP (Logit(*p*) = 2.619 − 0.809 × serum MFG-E8 + 0.0226 × serum DCP) distinguished HCC patients from CH and LC patients with an area under the curve of 0.923, a sensitivity of 81.1%, and a specificity of 89.8%. Futhermore, low preoperative serum MFG-E8 was an independent predictor of poor overall survival. Thus, serum MFG-E8 could serve as a feasible diagnostic and prognostic biomarker for HCC.

## Introduction

Hepatocellular carcinoma (HCC) is the fifth most common malignancy and the second-leading cause of cancer mortality worldwide^1,2^. HCC incidence and mortality have been increasing in recent decades. Three biomarkers, α-fetoprotein (AFP), lens culinaris agglutinin-reactive fraction of α-fetoprotein (AFP-L3), and des-γ-carboxy prothrombin (DCP), are used for HCC surveillance and diagnosis in parallel with imaging^3,4^. However, these markers are insufficient for early diagnosis of small HCCs. Hepatic resection of HCC is a curative treatment for patients who have optimal characteristics as defined by the Barcelona Clinic Liver Cancer staging system^5^. However, the recurrence rate after curative resection is over 10% within one year post-surgery and reaches 70–80% 5 years post-surgery^6,7^. Early HCC recurrence (within 1 year) is a critical determinant conferring poor prognosis^8,9^. Pathological factors, such as microscopic vascular invasion and intrahepatic metastasis are related to postoperative recurrence and prognosis^9,10^, and serum biomarkers complementing them are highly useful clinically. Thus, serum biomarkers for detecting HCC or predicting postoperative prognosis are in great demand in clinical practice.

Milk fat globule-epidermal growth factor 8 (MFG-E8; also known as lactadherin) is ubiquitously expressed on various organs and cells^11^, and acts as a bridging molecule between phosphatidylserine (PS) on apoptotic cells and α_v_β_3_ or α_v_β_5_ integrins on phagocytes^12,13^. Due to accumulation of unengulfed apoptosis cells, MFG-E8 knock-out mice developed autoimmune diseases^13^ or exacerbated inflammatory responses and a substantial decrease in survival following myocardial infarction^14^.

MFG-E8 also acts as a bridging molecule between PS on extracellular vesicles (EVs) such as exosomes, microvesicles, and microparticles^15–18^ and integrins α_v_β_3_ or α_v_β_5_ on various cells^16^. EVs are membrane vesicles (approximately 120 nm in size) derived from the endocytic compartment of the cell and play important roles in intercellular communication by exchanging proteins, lipids, mRNAs, and microRNAs^17^. EVs secreted from cancer cells can suppress the anti-tumor functions of immune cells, promote cancer metastasis, and cause drug resistance^19^.

An SY *et al*. recently reported that MFG-E8 secreted by mesenchymal stem cells strongly inhibited TGF-β signaling by binding to the α_v_β_3_ integrin on activated hepatic stellate cells, resulting in protection from liver fibrosis^20^. They also showed that expression of MFG-E8 was decreased in cirrhotic livers, but serum MFG-E8 levels were comparable in patients with liver cirrhosis and healthy controls^20^. The significance of MFG-E8 in patients with HCC has yet to be clarified.

In this study, we evaluated serum MFG-E8 levels and their association with EVs in patients with HCC. Our findings suggest that MFG-E8 exists with EVs in the circulation. Serum levels of MFG-E8 could feasibly serve as a biomarker for detecting HCC in HVs, CH patients and LC patients, and for predicting early HCC recurrence in patients who underwent curative liver resection.

## Results

### Serum MFG-E8 levels were significantly lower in HCC patients

**Figure 1:**
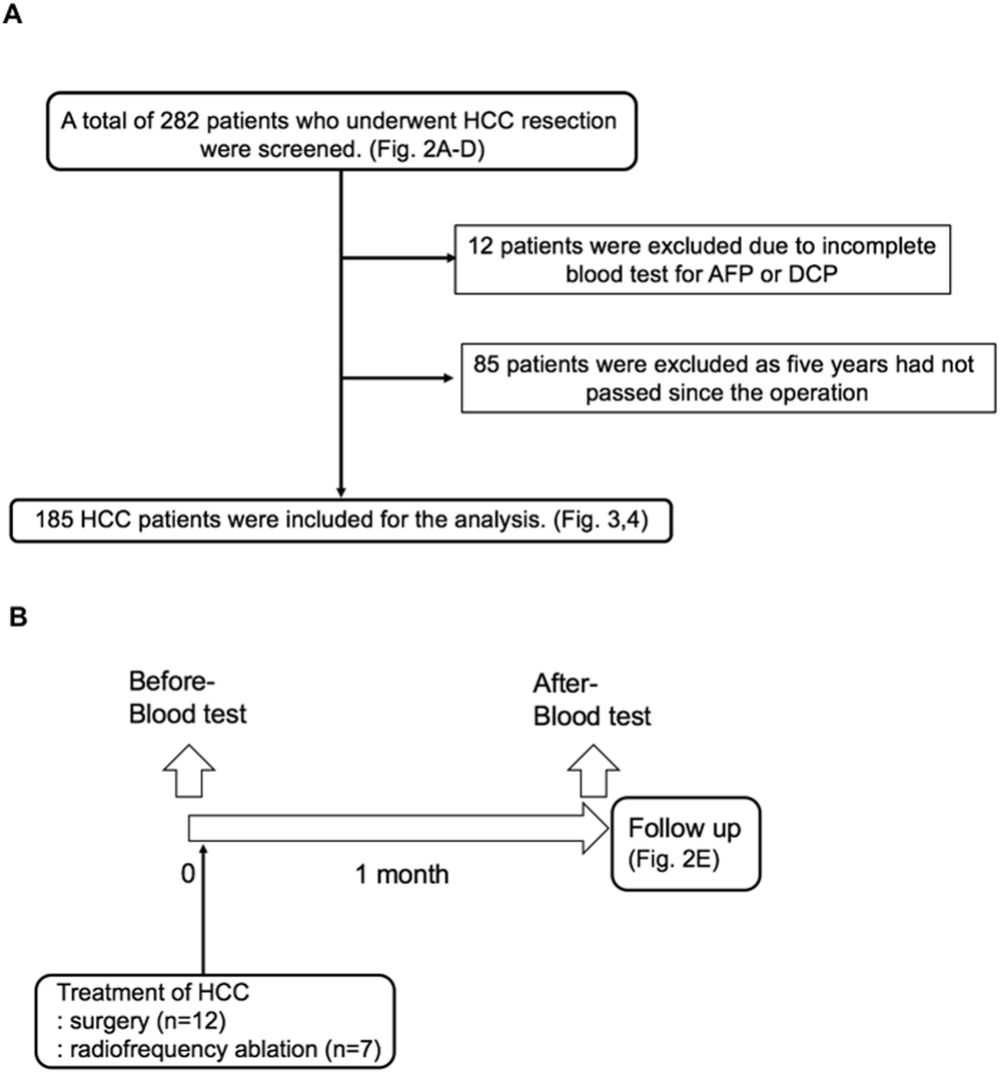
Flowchart of HCC patients selection process. (**A**) Retrospective study. (**B**) Prospective study. HCC, hepatocellular carcinoma; AFP, α-fetoprotein; DCP, des-γ-carboxy prothrombin.

**Figure 2:**
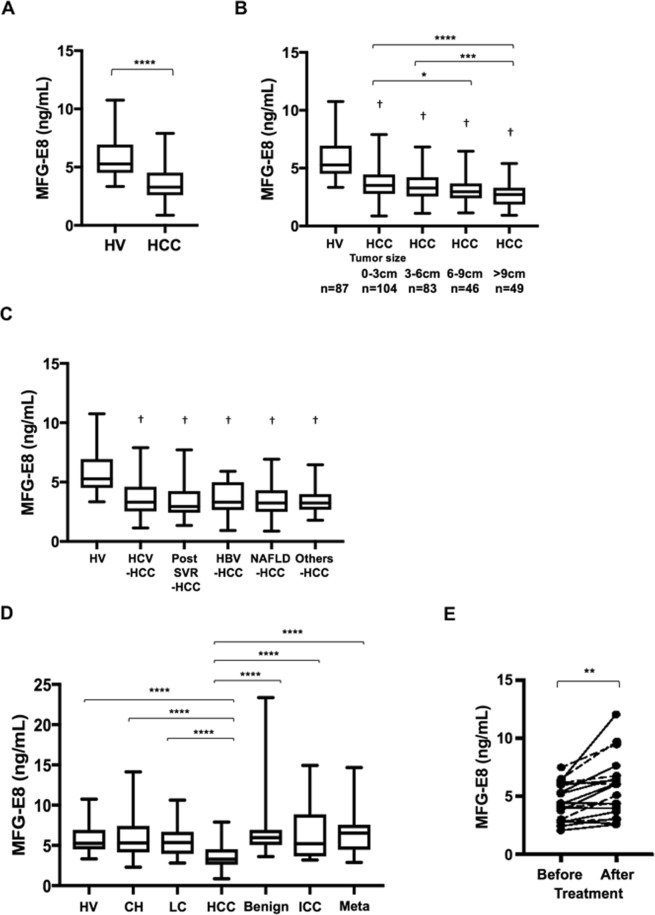
Serum MFG-E8 levels in patients with liver diseases. Serum milk fat globule-EGF factor 8 (MFG-E8) levels are shown for healthy volunteers (HVs) and hepatocellular carcinoma (HCC) patients (**A**), for HCC patients stratified by tumor size (**B**) by etiology of liver diseases (**C**), for hepatitis B virus (HBV)-infected, hepatitis C virus (HCV)-infected, post-sustained virologic response (SVR) and non-alcoholic fatty liver disease (NAFLD) patients with chronic hepatitis (CH), liver cirrhosis (LC), and HCCs (**D**), and in patients with benign liver tumors (Benign), intrahepatic cholangiocarcinoma (ICC), or metastatic liver tumors derived from primary colon cancer (Meta) (**D**) and for HCC patients before and after surgery (solid line, n = 12) or radiofrequency ablation (dashed line, n = 7) (**E**). Box plots represent the interquartile range and whiskers show minimum and maximum values. The line in each box shows the median. *p < 0.05, **p < 0.01, ***p < 0.001, ****p < 0.0001, ^†^p < 0.0001 compared with HV by Kruskal-Wallis test with Dunn’s multiple comparison test. Paired Student’s t test was used for the analysis presented in (**E**).

The flowcharts depicting the HCC patients selection process in this research were shown in Fig. 1A,B. Serum MFG-E8 levels were significantly lower in HCC patients (n = 282) than in HVs (n = 87) (3.6 ± 0.1 vs 5.8 ± 0.2 ng/mL, p < 0.0001) (Fig. 2A). Intriguingly, serum MFG-E8 levels were significantly decreased even in patients with a single HCC smaller than 3 cm (Fig. 2B). Serum MFG-E8 levels were lower in patients with larger HCCs (Fig. 2B). Serum MFG-E8 levels were decreased in HCC patients regardless of etiology (Fig. 2C). In patients with hepatitis C virus (HCV) infection, hepatitis B virus (HBV) infection and nonalcoholic fatty liver disease (NAFLD), serum MFG-E8 levels were comparable among HVs, CH patients and LC patients (Fig. 2D). In addition, serum MFG-E8 levels were not decreased in patients with liver benign tumors, cholangiocarcinoma, or liver metastasis (Fig. 2D). Moreover, serum MFG-E8 levels recovered after treatment of HCC by surgery (n = 12) or radiofrequency ablation (n = 7) (p = 0.0017) (Fig. 2E). These results showed that serum MFG-E8 levels were specifically decreased only in patients with HCC.

### Serum MFG-E8 levels could discriminate HCC patients from chronic hepatitis and liver cirrhosis patients

**Figure 3:**
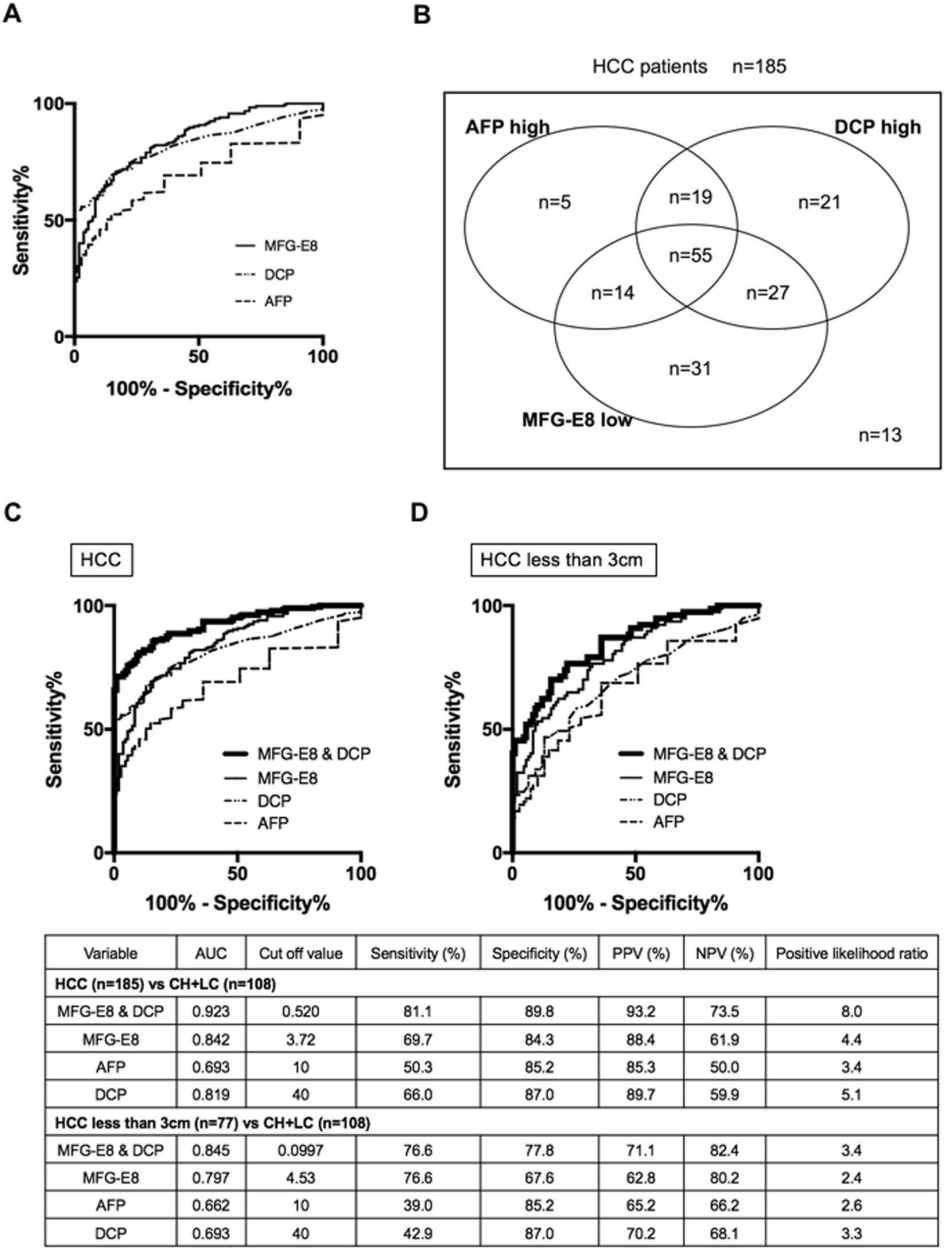
ROC analyses of serum MFG-E8, AFP, and DCP levels and our prediction model for diagnosis of HCC patients. (**A**) Receiver operating characteristic (ROC) curves for distinguishing hepatocellular carcinoma (HCC) patients (n = 185) from chronic hepatitis (CH)/ liver cirrhosis (LC) patients (n = 108). The optimal cutoff value for milk fat globule-EGF factor 8 (MFG-E8) was determined as those yielding the minimal value for (1 − sensitivity)^2^ + (1 − specificity)^2^. (**B**) Venn diagram presenting the distribution of HCC patients according to MFG-E8, α-fetoprotein (AFP), and des-γ-carboxy prothrombin (DCP). (**C,D**) Performance assessment of our prediction model and other HCC tumor biomarkers for HCC for distinguishing HCC patients (n = 185) from CH/LC patients (n = 108) (**C**), and patients with HCCs smaller than 3 cm (n = 77) from CH/LC patients (n = 108) (**D**). AUC, area under the curve; PPV, positive predictive value; NPV, negative predictive value.

We evaluated the feasibility of using serum MFG-E8 levels as a diagnostic biomarker for HCC. The cutoff values of AFP and DCP were defined as 10 ng/ml and 40 mAU/ml respectively according to each facility standard^21^. Serum MFG-E8 levels discriminated HCC patients (n = 185) from CH and LC patients (n = 108) with an area under the curve (AUC) of 0.842, a sensitivity of 69.7%, and a specificity of 84.3% (Fig. 3A). Serum MFG-E8 provided superior predictive ability for HCC compared with AFP and DCP (Fig. 3A). Among 185 patients, 44 patients were negative for both AFP and DCP. Seventy percents of AFP-negative and DCP-negative HCC patients (31/44) showed low MFG-E8 levels (Fig. 3B). From these results, serum MFG-E8 could be a novel serum tumor marker for HCC.

### Developing a new HCC diagnostic model using serum MEG-E8 and DCP

Next, we established a better predictive model for HCC diagnosis by stepwise multiple logistic regression analysis as follows.

$${\rm{Logit}}\,(p)=2.619-0.809\times {\rm{serum}}\,{\rm{MFG}}\,-\,{\rm{E}}8+0.0226\times {\rm{serum}}\,{\rm{DCP}}$$

The AUC, sensitivity, and specificity of our prediction model were 0.923, 81.1% and 89.8%, respectively, for HCC (Fig. 3C), and 0.845, 76.6%, and 77.8%, respectively, for small HCCs less than 3 cm (Fig. 3D). This prediction model, based on serum MFG-E8 and DCP levels, could help clinicians diagnose HCC earlier and more accurately.

### Low serum MFG-E8 level was associated with early recurrence in postoperative HCC patients

We also examined the relationship between preoperative serum MFG-E8 levels and HCC recurrence or survival following hepatectomy in 185 HCC patients who underwent curative hepatic resection. Patients were observed for at least 5 years after initial curative resection (Table 1). Among this cohort, 106 patients (57%) experienced a recurrence within 5 years. We then sub-categorized patients as follows: patients who had a recurrence within 1 year were the early recurrence (ER) group (n = 54, 29%)^9^, those who had a recurrence within 1–5 years of resection were the late recurrence (LR) group (n = 52, 28%) and patients whose disease did not recur were the no recurrence (NR) group (n = 79, 43%) (Supplementary Table 1).

**Table 1:** Correlation between serum MFG-E8 level and clinicopathological features in 185 HCC patients.

Variable	All cases (n = 185)	High MFG-E8 group (n = 67)	Low MFG-E8 group (n = 118)	*P value*
Gender (male/female)	142/43	51/16	91/27	0.88
Age (years)^##^	68.2 ± 0.8	68.4 ± 1.3	68.0 ± 1.0	0.80
HBV/HCV/NBNC(NASH/NAFLD)	29/66/95 (83)	14/27/28 (21)	15/39/67 (62)	0.21
Child-Pugh score A/B/C	177/8/0	65/2/0	112/6/0	0.50
Differentiation (well and moderate/poor)	135/50	49/18	86/32	0.97
Tumor size (cm)^##^	5.4 ± 0.3	4.5 ± 0.5	5.9 ± 0.4	0.03
Vascular invasion (vp, vv, va) (yes/no)	69/116	24/43	45/73	0.75
pStage I/II/III/IV	83/31/71/0	34/13/20/0	49/18/51/0	0.20
AFP (ng/ml)^#^	10.3 (3.6–60.7)	7 (3.5–48.9)	13.2 (4.4–230.0)	0.72
DCP (mAU/ml)^#^	117 (24–1862)	135 (32–852)	204 (26–3775)	0.24
Serum albumin (g/dl)^##^	4.0 ± 0.1	4.0 ± 0.1	3.9 ± 0.1	0.36
Total bilirubin (mg/dl)^##^	0.8 ± 0.1	0.8 ± 0.1	0.7 ± 0.1	0.29
ICG test (%)^##^	14.7 ± 0.7	15.7 ± 1.2	14.2 ± 0.9	0.31
Platelet (×10^4^/μl)^##^	17.0 ± 0.6	17.0 ± 0.9	17.0 ± 0.5	0.95
AST (IU/l)^##^	44 ± 2	49 ± 4	42 ± 3	0.12
ALT (IU/l)^##^	37 ± 2	41 ± 3	34 ± 3	0.11
Cr (mg/dl)^##^	0.8 ± 0.1	0.8 ± 0.1	0.7 ± 0.1	0.08

MFG-E8, milk fat globule-EGF factor 8; HBV, hepatitis B virus; HCV, hepatitis C virus; NBNC, non-B non-C hepatitis; NASH, non-alcoholic steatohepatitis; NAFLD, non-alcoholic fatty liver disease; vp, portal vein invasion; vv, hepatic vein invasion; va, hepatic artery invasion; pStage, pathologic stage by the eighth edition of the UICC TNM classification; AFP, α-fetoprotein; DCP, des-γ-carboxy prothrombin; ICG, indocyanine green; AST, aspartate transaminase; ALT, alanine aminotransferase; Cr, creatinine.

The low and high MFG-E8 groups were separated by the cut-off value of 3.42.

^#^Data displayed as median (25th to 75th percentile). ^##^All other values were expressed as mean ± standard error.

**Figure 4:**
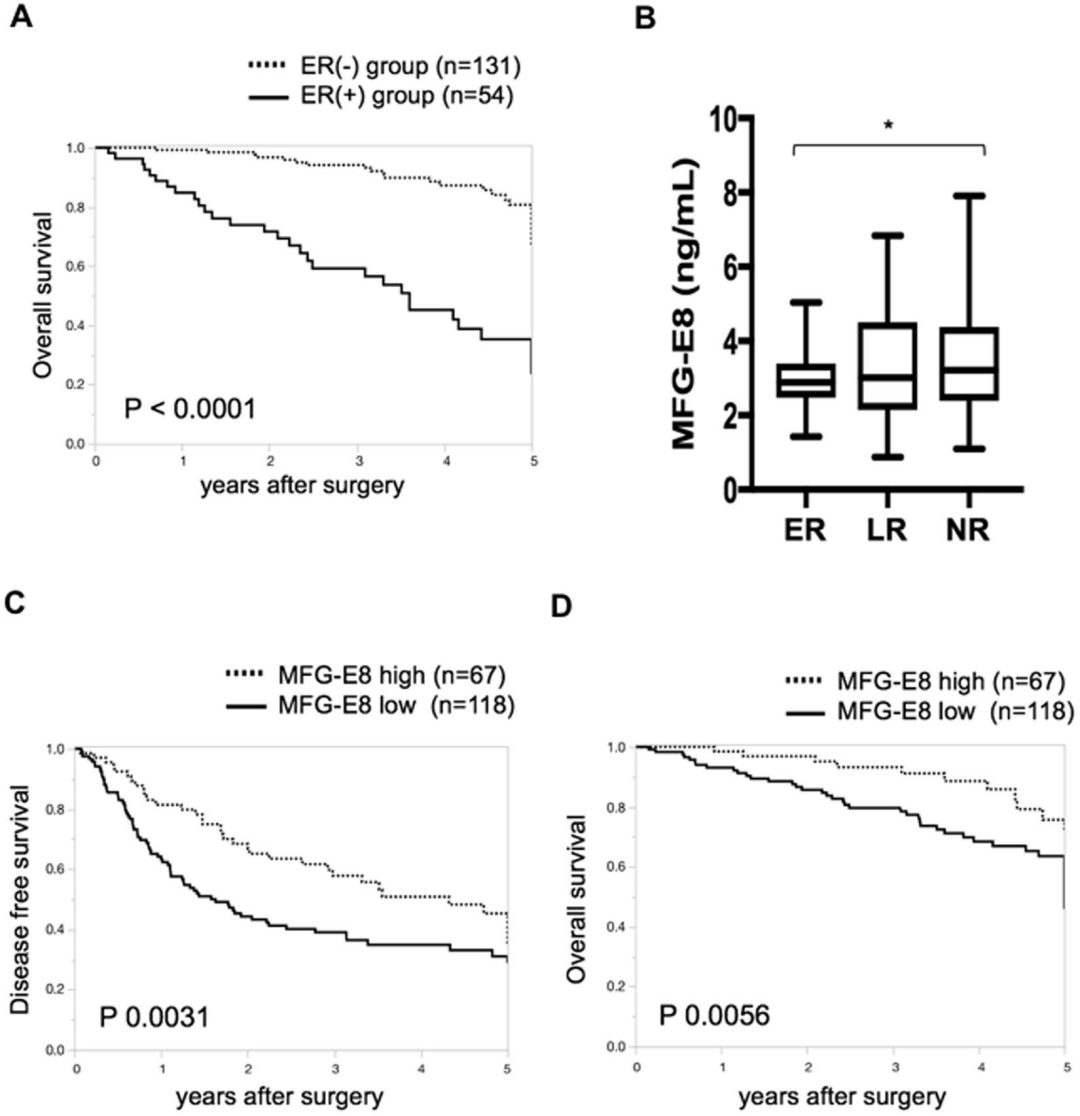
The relationship between serum preoperative MFG-E8 levels and HCC recurrence or survival following hepatectomy. (**A**) Kaplan–Meier analysis of overall survival (OS) for patients stratified by early recurrence (ER) within 1 year of resection (n = 54) and no recurrence within 1 year of resection (n = 131). (**B**) Serum milk fat globule-EGF factor 8 (MFG-E8) levels for preoperative hepatocellular carcinoma (HCC) patients grouped by early recurrence (ER; n = 54), late recurrence (LR; n = 52), and no recurrence (NR; n = 79). (C,D) Kaplan–Meier analysis of disease free survival (DFS) (**C**) and overall survival (OS) (**D**) for HCC patients stratified by serum MFG-E8 levels (cut-off: 3.42 ng/mL). Box plots show the interquartile range and whiskers show the minimum and maximum values. The line in each box shows the median. *p < 0.05.

The ER group was associated with unfavorable overall survival (OS) compared with the LR and NR groups (p < 0.0001; Fig. 4A). Serum MFG-E8 levels were significantly lower in the ER group than in the NR group (ER group 3.0 ± 0.1 vs NR group 3.5 ± 0.2 ng/mL, p = 0.0324; Fig. 4B). The optimal cut-off point for serum MFG-E8 to classify our subjects into the ER or LR/NR groups on the receiver operating characteristic (ROC) curve was 3.42 ng/mL. We then compared the clinical parameters between low and high serum MFG-E8 patients (divided by the cut-off at 3.42). Tumor size showed the only significant difference between the groups (Table 1). Neither etiology nor histology was associated with preoperative serum MFG-E8 levels. Univariate and multivariate analysis confirmed that low serum MFG-E8 levels and large tumor size were independently associated with ER (Table 2).

**Table 2:** Factors associated with early recurrence after hepatectomy for HCC (n = 185).

Variable	Univariate analysis			Multivariate analysis		
	OR	95% CI	*P value* ^a^	OR	95% CI	*P value* ^b^
Gender (male/female)	0.92	0.43–1.97	0.83			
Age (years)	1.00	0.97–1.03	0.87			
HBV/HCV/NBNC(NASH/NAFLD)	—	—	0.11			
Child-Pugh score A/B/C	1.48	0.34–6.43	0.60			
Differentiation (well and moderate/poor)	0.50	0.25–1.00	0.05			
Tumor size (cm)	1.18	1.09–1.27	<0.0001	1.14	1.05–1.25	<0.01
Vascular invasion (vp, vv, va) (yes/no)	2.64	1.38–5.06	<0.01	1.75	0.84–3.64	0.13
AFP (ng/ml)	1.00	0.99–1.00	0.21			
DCP (mAU/ml)	1.00	0.99–1.00	0.27			
Serum albumin (g/dl)	0.52	0.26–1.04	0.06			
Total bilirubin (mg/dl)	0.56	0.20–1.54	0.25			
ICG test (%)	1.01	0.98–1.05	0.37			
Platelet (×10^4^/μl)	1.01	0.99–1.03	0.37			
AST (IU/l)	1.00	0.99–1.01	0.97			
ALT (IU/l)	0.98	0.97–1.00	0.06			
Cr (mg/dl)	0.41	0.03–5.90	0.51			
Low MFG-E8 group (yes/no)	2.53	1.22–5.25	0.01	2.22	1.03–4.79	0.04

^a^χ^2^ test; ^b^Logistic regression analysis.

OR, odds ratio; CI, confidence interval; HBV, hepatitis B virus; HCV, hepatitis C virus; NBNC, non-B non-C hepatitis; NASH, non-alcoholic steatohepatitis; NAFLD, non-alcoholic fatty liver disease; vp, portal vein invasion; vv, hepatic vein invasion; va, hepatic artery invasion; AFP, α-fetoprotein; DCP, des-γ-carboxy prothrombin; ICG, indocyanine green; AST, aspartate transaminase; ALT, alanine aminotransferase; Cr, creatinine; MFG-E8, milk fat globule-EGF factor 8.

The low and high MFG-E8 groups were separated by the cut-off value of 3.42.

### Preoperative serum MFG-E8 was an independent predictor of DFS and OS in HCC patients who underwent hepatic resection

Low serum MFG-E8 levels (cut-off 3.42) were also associated with unfavorable disease free survival (DFS) and OS (Fig. 4C,D). Univariate and multivariate analysis indicated that low serum MFG-E8 levels and large tumor size were independently associated with poor OS (Table 3). These results showed that serum MFG-E8 level can feasibly serve as a biomarker for early recurrence and poor prognosis in HCC patients undergoing initial hepatic resection.

**Table 3:** Factors associated with overall survival after hepatectomy for HCC (n = 185).

Variable	Univariate analysis			Multivariate analysis		
	HR	95% CI	*P value* ^c^	HR	95% CI	*P value* ^d^
Gender (male/female)	1.53	0.69–3.38	0.29			
Age (years)	1.00	0.97–1.03	0.97			
HBV/HCV/NBNC(NASH/NAFLD)	—	—	0.60			
Child-Pugh score A/B/C	0.44	0.16–1.83	0.22			
Differentiation (well and moderate/poor)	0.59	0.30–1.18	0.14			
Tumor size (cm)	1.20	1.11–1.30	<0.0001	1.16	1.06–1.27	<0.01
Vascular invasion (vp, vv, va) (yes/no)	2.02	1.06–3.85	0.03	1.03	0.48–2.26	0.92
AFP (ng/ml)	1.00	0.99–1.00	0.68			
DCP (mAU/ml)	1.00	1.00–1.00	0.02	1.00	0.99–1.00	0.17
Serum albumin (g/dl)	0.64	0.33–1.27	0.20			
Total bilirubin (mg/dl)	0.57	0.21–1.58	0.27			
ICG test (%)	1.02	0.99–1.06	0.14			
Platelet (×10^4^/μl)	1.01	0.99–1.03	0.43			
AST (IU/l)	1.00	0.99–1.01	0.67			
ALT (IU/l)	1.00	0.98–1.01	0.57			
Cr (mg/dl)	0.11	0.01–2.54	0.16			
Low MFG-E8 group (yes/no)	3.03	1.44–6.38	<0.01	2.59	1.18–5.70	0.02

^c^Log-rank test; ^d^Cox proportional hazards model.

HR, hazard ratio; CI, confidence interval; HBV, hepatitis B virus; HCV, hepatitis C virus; NBNC, non-B non-C hepatitis; NASH, non-alcoholic steatohepatitis; NAFLD, non-alcoholic fatty liver disease; vp, portal vein invasion; vv, hepatic vein invasion; va, hepatic artery invasion; AFP, α-fetoprotein; DCP, des-γ-carboxy prothrombin; ICG, indocyanine green; AST, aspartate transaminase; ALT, alanine aminotransferase; Cr, creatinine; MFG-E8, milk fat globule-EGF factor 8.

The low and high MFG-E8 groups were separated by the cut-off value of 3.42.

### Serum EVs isolated by the Tim4 affinity method were significantly lower in HCC patients

**Figure 5:**
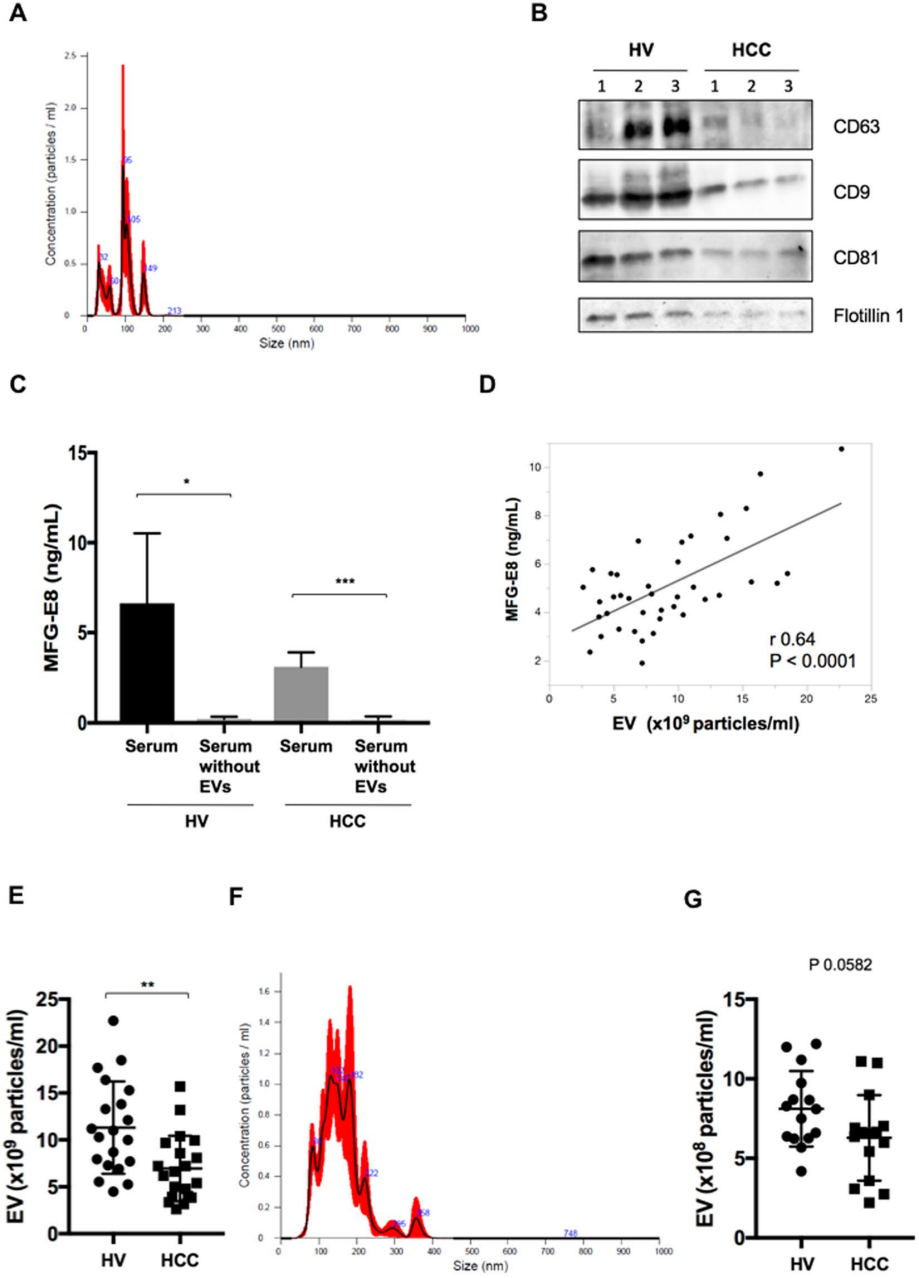
Serum MFG-E8 levels and EVs in HVs and HCC patients. (**A**) Extracellular vesicles (EVs) isolated using the Tim4 affinity method were analyzed by nanoparticle tracking analysis (NTA) using NanoSight LM10. (**B**) Expression of the EV markers CD63, CD9, CD81 and flotillin 1 by western blotting. Full-length blots are presented in Supplementary Fig. 2. (**C**) Comparision of serum milk fat globule-EGF factor 8 (MFG-E8) levels before and after EV isolation by the Tim4 affinity method. (**D**) Correlation between serum MFG-E8 levels and the concentration of serum EVs isolated using the Tim4 affinity method. Sera were drawn from healthy volunteers (HVs) (n = 20) and hepatocellular carcinoma (HCC) patients (n = 20). (**E**) Comparative analysis of serum EVs from HVs and HCC patients by NTA. (**F**) EVs isolated using the ultracentrifugation method were analyzed by NTA using NanoSight LM10. (**G**) Comparative analysis of serum EVs isolated by the ultracentrifugation method from HCC patients (n = 15) and HVs (n = 15). *p < 0.05, **p < 0.01, ***p < 0.001 by Kruskal-Wallis test with Dunn’s multiple comparison test.

We showed that serum MFG-E8 levels were decreased in HCC patients compared with HVs and that these levels recovered after curative hepatic resection or ablation. However, the mechanism of serum MFG-E8 reduction in HCC patients remained unknown. First, we assessed the presence of apoptotic cells in HCC tissues. TUNEL-positive cells could almost not be identified and apoptotic cells were absent in HCC tissues (data not shown). Next, we hypothesized that MFG-E8 bound to EVs in the circulation because EVs expose phosphatidylserine (PS). Serum samples (500 μL) were obtained from HCC patients (n = 20) and HVs (n = 20). EVs, including exosomes and microvesicles, were isolated from each serum sample using the MagCapture Exosome Isolation Kit PS^22^. EVs isolated from these sera were characterized by nanoparticle tracking analysis (NTA) (Fig. 5A) and showed high concentrations of the exosome protein markers CD63, CD9, CD81, and flotillin1 by western blotting (Fig. 5B and Supplementary Fig. 2A). Serum MFG-E8 levels became undetectable after EVs were depleted from sera of HVs and HCC patients by the Tim4 affinity method (HVs: p = 0.0425, HCC patients: p = 0.0002; Fig. 5C). Moreover, serum MFG-E8 levels and the concentrations of serum EVs showed a positive correlation (r = 0.64, p < 0.0001, Fig. 5D). These data suggested that most MFG-E8 was bound to PS expressed on the surface of EVs in sera. Next, we compared the quantity of EVs in HVs and HCC patients. EV concentrations were lower in HCC patients than in HVs (7.0 ± 0.8 vs 11.3 ± 1.1 × 10^9^ particles/ml, p = 0.0026; Fig. 5B,E). When we used the ultracentrifugation method for isolating EVs and the size distribution and concentration of EVs were analyzed by NTA (Fig. 5F), EV concentrations also tended to be lower in HCC patients than in HVs (6.3 ± 0.7 vs 8.1 ± 0.6 × 10^8^ particles/ml, p = 0.0582; Fig. 5G). Based on the above-mentioned EVs experiments, we concluded that most MFG-E8 was associated with EVs in the circulation.

## Discussion

The diagnosis of HCC patients remains difficult, especially in the early stages of the disease. The overall 5-year survival rate of HCC is still very low, partly due to the unsatisfactory predictive power of conventional HCC biomarkers (e.g., AFP, AFP-L3, and DCP)^23^. Multi-marker prediction algorithms would contribute toward distinguishing HCC from non-malignant chronic liver diseases and may be useful for the surveillance of cirrhosis^24,25^. In the present study, serum MFG-E8 level was superior to AFP or DCP in distinguishing HCC patients from CH/LC patients (Fig. 3A). Using stepwise multiple logistic regression analysis, we further constructed a prediction model for HCC diagnosis, combining serum MFG-E8 and DCP levels, which showed enhanced diagnostic performance. Our prediction model might be useful for the surveillance for HCC in CH and LC patients and contribute to prolonged OS.

Splitting training and validation sets in the subjects was one of the options, however, the sample size of our study was not enough (HCC, n = 185; CH + LC, n = 108). Therefore, in order to secure the estimation accuracy of the prediction model, we used all data to construct the model for HCC diagnosis. Our prediction model for HCC needs to be validated further in the other set of patients.

Furthermore, we found that serum MFG-E8 level could predict the prognoses of HCC patients who underwent liver curative resection. Liver resection is recommended as a promising treatment option for early-stage HCC^26^, but common postoperative recurrence still leads to unsatisfactory outcomes^27,28^. Many risk factors are reported to influence postoperative recurrence of HCC, such as tumor size, tumor number, staging, vascular invasion, pathologic differentiation, serum AFP level, liver function reserve, and hepatitis viral status^29–32^. We show here that serum MFG-E8 contributed to relapse after HCC resection and life prognosis, independently from these previously reported factors. MFG-E8 could play an important role in the pathogenesis of HCC.

Alteration of serum MFG-E8 levels in relation to clinical manifestations has been reported in rheumatoid arthritis (RA), type 2 diabetes, and systemic lupus erythematosus (SLE) patients. Patients with RA have lower serum concentrations of MFG-E8 compared with HVs and serum MFG-E8 levels are normalized after treatment^33^. Patients with type 2 diabetes also have lower serum concentrations of MFG-E8, which decrease further over the course of disease progression^34^. In contrast, in SLE patients, serum MFG-E8 levels are higher than those of HVs^35^. The pathophysiology underlying regulation of serum MFG-E8 levels in these disease conditions is largely unknown. In the present study, we showed that serum MFG-E8 levels were significantly decreased in HCC patients.

One of the biggest limitations of this study was the unclear mechanism of the reduction of MFG-E8 in patients with HCC compared to patients without HCC. We assume three possibilities: (i) the presence of HCC diminishes production of MFG-E8, (ii) HCC enhances the uptake of MFG-E8 into some cells, and/or (iii) HCC promotes the degradation of MFG-E8.

MFG-E8 expression in cirrhotic liver tissues is profoundly reduced compared with normal liver tissues, while serum levels of MFG-E8 are comparable in HVs and LC patients^20^. The expression levels of MFG-E8 were higher in the cancerous liver tissues than those in noncancerous liver tissues (Supplementary Figs 1A–C and 2B) although serum levels of MFG-E8 were decreased in HCC patients compared with HVs. There was no correlation between serum MFG-E8 and the expression levels of MFG-E8 mRNA in both cancerous and noncancerous liver tissues (Supplementary Fig. 1D,E). Therefore, hepatocytes and HCC cells might not be the main producers of serum MFG-E8.

In relation to the second hypothesis, enhanced clearance of MFG-E8 by phagocytes or HCC cells might be possible. We showed here that serum MFG-E8 levels are positively correlated with concentrations of EVs in the sera. The same amount of EVs from HCC patients and HV showed comparable MFG-E8 levels (p = 0.6551). Depletion of EVs from patient sera reduced MFG-E8 to almost undetectable levels, indicating that EVs and MFG-E8 are associated in the circulation, and serum MFG-E8 could be a signature of serum EV levels. EVs such as exosomes and microvesicles expose PS on their surface^36^. MFG-E8 binds to PS on the surface of EVs through its carboxy-terminal factor V/VIII-like domain, linking EVs to α_v_β_3_ integrin- or α_v_β_5_ integrin-expressing phagocytes^12,37,38^. α_v_β_5_ integrins are reported to be highly expressed in HCC^39–41^. Taking these findings and reports into consideration, it is possible that EVs and MFG-E8 are jointly involved in adherence to α_v_β_5_ integrins on HCCs. Nakaya *et al*. reported that cardiac myofibroblasts not only produced MFG-E8, but also engulfed dead cells via a MFG-E8/integrin α_v_β_5_–dependent pathway and attenuated inflammation after myocardial infarction^14^. Another candidate for the cells engulfing EVs are cancer-associated fibroblasts, which are reported to express integrin α_v_β_5_.

In relation to the third hypothesis, considering the association of MFG-E8 with EVs, it is plausdible that significant decreases of serum MFG-E8 in HCC patients is the consequence of lesser concentrations of circulating EVs. The metabolic pathway of MFG-E8 in the body, either in its free or EV-bound form, has not been well understood. Negligible amounts of free MFG-E8 in patient serum implies a different turn-over rate of MFG-E8 with or without associated EVs. Phagocytes and macrophages are presumably the main cell population responsible for the degradation of MFG-E8. Further investigation may be needed to clarify whether phagocytic activity is accelerated or not in patients with HCC.

In summary, we showed here that serum MFG-E8 serves as an independent diagnostic as well as prognostic biomarker for HCC patients. Using a prediction model including MFG-E8 and DCP level, the diagnostic ability for HCC was even more effective than MFG-E8 alone. Serum MFG-E8 could feasibly be used to diagnose patients with small HCC, thus giving clinicians advantages to obtain early diagnosis. Serum MFG-E8 could be also used to predict early recurrence and overall survival before hepatic resection, thus helping clinicians coordinate post-operative follow-up strategies.

## Methods

### Study subjects

To validate the feasibility of using serum MFG-E8 levels as a biomarker for HCC, we enrolled 282 HCC patients [HBV (n = 30), HCV (n = 68), post-sustained viral response (SVR) to HCV (n = 25), NAFLD (n = 138), others who excluded patients with HBV, HCV, NAFLD, alcohol abuse, primary biliary cirrhosis, primary sclerosing cholangitis, hemochromatosis, Wilson disease, and autoimmune hepatitis (n = 21)] (Fig. 1A), 108 patients with chronic hepatitis (CH) (n = 54, HBV/HCV/NAFLD: 18/18/18) and liver cirrhosis (LC) (n = 54, HBV/HCV/NAFLD: 18/18/18), nine patients with benign liver tumors (focal nodular hyperplasia, hemangioma, angiomyolipoma, or neuroendocrine tumor), 18 patients with intrahepatic cholangiocarcinoma, 52 patients with metastatic liver tumors arising from colon cancer, and 87 healthy volunteers (HVs). The patients were followed at Kyushu University Hospital, Hokkaido University Hospital, and the Kohnodai Hospital, National Center for Global Health and Medicine, between 2001 and 2018. Serum samples were collected before hepatic resection or radiofrequency ablation and stored at −80 °C. Non-HCC conditions were diagnosed independently according to the Brunt system by pathologists at each facility. Diagnosis of CH and LC was made from liver biopsies in patients undergoing surgery or by imaging [ultrasound (US), computed tomography (CT) or magnetic resonance imaging (MRI)] for patients treated by internal medicine. The absence of HCC was confirmed using serum tumor markers including AFP, AFP-L3, and DCP and by US, CT, or MRI. Pathological diagnosis of resected tissues was performed by an independent pathologist. We excluded HCC patients who underwent non-curative resection, defined as cases in which positive cancer cells were identified at the resected margin. Patients with human immunodeficiency virus infection were excluded. HVs had no history of liver diseases, malnutrition, malignant tumors, autoimmune diseases or other severe morbidities.

To evaluate whether serum MFG-E8 could be used as a novel tumor marker for HCC and whether serum MFG-E8 levels were associated with recurrence or survival following hepatectomy, we analyzed 185 patients who underwent primary curative hepatic resection for HCC with complete blood test for both AFP and DCP, and who had been observed for more than 5 years after initial curative resection (Fig. 1A). Curative resection was defined as complete macroscopic removal of the tumor. After the operation, patients were followed-up through hospital visits at 3-month intervals during which liver function, AFP, enhanced CT and/or enhanced MRI tests were performed. Early recurrence after curative resection was defined as intrahepatic, regional or systemic recurrence within 1 year^9^.

In addition, as a prospective study, we enrolled 19 patients treated by surgery (n = 12) or by radiofrequency ablation (n = 7) and evaluated the changes in serum MFG-E8 with treatment (Fig. 1B).

The study protocol conformed to the ethical guidelines for human clinical research established by the Japanese Ministry of Health, Labor and Welfare and was approved by the ethics committee at Kyushu University, Hokkaido University and the National Center for Global Health and Medicine. Written informed consent was obtained from all patients at enrollment.

### Enzyme-linked immunosorbent assay (ELISA)

Serum MFG-E8 levels were measured using an ELISA kit (DFGE80; R&D Systems, Minneapolis, MN, USA) according to the manufacturer’s instructions.

### Serum extracellular vesicle (EV) isolation

100 or 500 μL of serum samples (for the Tim4 affinity method or ultracentrifugation methods, respectively) was centrifuged for 5 min at 300 × *g* at 4 °C to remove cells. The supernatants were transferred to new tubes and centrifuged for 20 min at 2,000 × *g* at 4 °C to remove cellular debris. Then, the supernatants were transferred to new tubes and centrifuged for 30 min at 10,000 × *g* at 4 °C to remove large EVs. The supernatants were passed through 0.22-μm filter units to completely remove larger EVs, and then the filtrate was subjected to further EV purification by the Tim4 affinity method or the ultracentrifugation method.

To isolate EVs such as exosomes by the Tim4 affinity method^42^, the MagCapture Exosome Isolation Kit PS (Fujifilm Wako, Japan) was used according to the manufacturer’s instructions^22^. EVs were isolated from 500 μL of serum. In brief, 0.6 mg of streptavidin magnetic beads loaded with 1 μg of biotinylated mouse Tim4-Fc was added to the filtered supernatant supplemented with 2 mM CaCl_2_ and the mixture was rotated overnight at 4 °C. The beads were washed three times with 1 mL of washing buffer (20 mM Tris-HCl, pH 7.4, containing 150 mM NaCl, 0.05% Tween-20 and 2 mM CaCl_2_) and bound EVs were eluted with elution buffer (8.1 mM Na_2_HPO_4_ and 1.47 mM KH_2_PO_4_, pH 7.4, containing 137 mM NaCl, 2.7 mM KCl, and 1 mM EDTA).

For the ultracentrifugation method, the filtered supernatants were diluted in sterile phosphate-buffered saline (PBS) to 5 mL in an ultracentrifugation tube and then centrifuged at 100,000 × *g* at 4 °C for 2 h (P55ST2 rotor K-50). The supernatants were carefully removed, sterile PBS were added and the centrifugation was repeated at 100,000 × *g* for 2 h. After the second centrifugation, the supernatants were carefully removed and the pellet was suspended in PBS by repeated pipetting. The samples were stored at 4 °C until quantification by nanoparticle tracking analysis (NTA).

### EVs size and concentration

The size distribution and concentration of EVs were measured by NTA using a NanoSight LM10 system (Malvern, UK) equipped with a fast video capture and particle tracking software. NTA post-acquisition settings were the same for all samples. Each video was analyzed to obtain the vesicle size and concentration. EV isolations and NTA analyses were performed blind. The isolated EVs were diluted 40-fold or 25-fold in PBS, then the size and concentration were measured three times per sample. Average values of size and concentration were shown. EVs were confirmed in the checklist as the ISEV guidelines for extracellular vesicles characterization^43^ (Supplementary Fig. 3).

### Western blotting

Purified EVs were lysed with 2× sodium dodecyl sulfate (SDS) sample buffer [100 mM Tris-HCl, pH 6.8, 4% (w/v) SDS, 20% (v/v) glycerol]. Total proteins were separated by SDS-PAGE, and then the following primary antibodies were used: anti-CD63 (mouse monoclonal, SHI-EXO-M02, 1:500, COSMO BIO Co. Ltd., Tokyo, Japan), anti-CD9 (mouse monoclonal, SHI-EXO-M01, 1:500, COSMO BIO Co. Ltd), anti-CD81 (mouse monoclonal, MA5-13548, 1:100, Thermo Fisher Scientific), anti-flotillin 1 (rabbit monoclonal, ab133497, 1:10,000, abcam). Subsequently, the following secondary antibodies were used: horseradish peroxidase (HRP)-conjugated anti-mouse IgG (NA931-1ML, 1:4,000, GE Healthcare UK Ltd, England), anti-rabbit IgG-HRP (NA934-1ML, 1:1,000, GE Healthcare UK Ltd). Protein bands were quantified by densitometry using Image Quant LAS 4000 (GE Healthcare UK Ltd, Amersham Place, Little Chalfont, Buckinghamshire HP7 9NA, England).

### Statistical analysis

Differences between two groups were assessed using the Mann–Whitney U test. Comparisons of the same individual were made using paired Student’s t tests. Multiple comparisons among more than two groups were made using Kruskal–Wallis and Dunn’s tests. The correlation between two groups was assessed by Spearman’s analysis. Associations among variables were determined by Fisher’s exact tests or χ^2^ tests. The diagnostic performance of the markers was assessed by analyzing receiver operating characteristic (ROC) curves. A prediction model for HCC was established using a stepwise multiple logistic regression analysis. A stepwise multivariate analysis was conducted to identify parameters that significantly contributed to the early recurrence and overall survival after hepatectomy for HCC.

Disease free survival (DFS) and overall survival (OS) curves were calculated using the Kaplan–Meier method and differences between groups were assessed using the log-rank test. Univariate and multivariate logistic regression analyses were used to identify independent determinants of early HCC recurrence and OS. Statistical analyses were performed using GraphPad Prism version 7.0 (GraphPad Software, San Diego, CA) and JMP Pro 13.0 (SAS Institute, Cary, NC, USA). P-values less than 0.05 were considered statistically significant.

## Supplementary information

Supplementary materials
