# Supplementary material for: Serum milk fat globule-EGF factor 8 (MFG-E8) as a diagnostic and prognostic biomarker in patients with hepatocellular carcinoma
Source: Sci Rep. 2019 Oct 31;9:15788. doi: 10.1038/s41598-019-52356-6 (PMC6823494; doi:10.1038/s41598-019-52356-6)
Supplement: Supplementary file 1 — Supplementary materials [file 41598_2019_52356_MOESM1_ESM.pdf]

Supplementary materials

Serum milk fat globule-EGF factor 8 (MFG-E8) as a diagnostic and prognostic biomarker in patients with hepatocellular carcinoma

Tomonari Shimagaki[1,2], Sachiyo Yoshio[1*], Hironari Kawai[1], Yuzuru Sakamoto[1], Hiroyoshi Doi[1], Michitaka Matsuda[1], Taizo Mori[1], Yosuke Osawa[1], Moto Fukai[3], Takeshi Yoshida[4], Yunfei Ma[4], Tomoyuki Akita[5], Junko Tanaka[5], Akinobu Taketomi[3], Rikinari Hanayama[4] , Tomoharu Yoshizumi[2], Masaki Mori[2], and Tatsuya Kanto[1*]

Methods

Immunohistochemistry

Patient samples were embedded in paraffin and cut into 5-μm-thick sections. Antigen retrieval was carried out in ANTIGEN UNMASKING SOLUTION (H-3301, Vector Laboratories, Burlingame, CA, USA), after deparaffinization and rehydration. Then, 3% H_2O_2 (Dako Corporation) was applied to block endogenous peroxidase. Mouse anti-human MFG-E8 monoclonal antibody, diluted 1:50 (sc-8029, Santa Cruz Biotechnology, Santa Cruz, CA, USA), was added to the samples, which were incubated at 4°C overnight. An EnVisionTM System (K4063, Dako, Carpinteria, CA, USA) was employed for immunohistochemical analysis the next day, and 3′3-diaminobenzidine (SK-4100, Vector Laboratories, Burlingame, CA, USA) was used as a chromogen for visualization. Cell nuclei were counterstained with haematoxylin. Images were photographed under the BZ-9000 Fluorescene Microscope (Keyence, Osaka, Japan).

RNA isolation and RT-PCR

Total RNA was isolated from cancerous and noncancerous liver tissues using the Maxwell® RSC simplyRNA Tissue Kit (Promega, Madison, WI, USA) according to the manufacturer's instructions. MFG-E8 expression levels were evaluated using RT-PCR and qPCR kits. Reverse transcription of total RNA into cDNA was performed with SuperScript® Ⅲ First-Strand Synthesis SuperMix (18080-400; invitrogen/Thermo Fisher Scientific, Carlsbad, CA, USA) according to the manufacturer's instructions. Transcripts were measured using TaqMan probes for MFG-E8 (Hs00983890_m1) and GAPDH (Hs02758991_g1) with TaqMan Gene Expression Master Mix (Applied Biosystems, Carlsbad, CA, USA), and subsequently analyzed on a Light Cycler® 480 System II (Roche).

Western blotting

Purified EVs were lysed with 2× sodium dodecyl sulfate (SDS) sample buffer [100 mM Tris-HCl, pH 6.8, 4% (w/v) SDS, 20% (v/v) glycerol]. Total protein from liver tissues was extracted in radioimmunoprecipitation assay (RIPA) buffer (89900, Thermo Fisher Scientific, Rockford, IL, USA). Protein concentrations were quantified by bicinchoninic acid (BCA) assay (5000112JA, Bio-Rad Laboratories, Hercules, CA, USA).

Total proteins were separated by SDS-PAGE, and then the following primary antibodies were used: anti-MFG-E8 (Mouse monoclonal, sc-8029, 1:200, Santa Cruz Biotechnology. Santa Cruz, CA, USA), anti-GAPDH (ab8245, 1:10,000, abcam. Cambridge, UK). Subsequently, the following secondary antibodies were used: horseradish peroxidase (HRP)-conjugated anti-mouse IgG (NA931-1ML, 1:4,000, GE Healthcare UK Ltd, England). Protein bands were quantified by densitometry using Image Quant LAS 4000 (GE Healthcare UK Ltd, Amersham Place, Little Chalfont, Buckinghamshire HP7 9NA, England) and normalized to GAPDH.

Supplementary Table

Supplementary Table 1. Clinicopathological parameters of 185 HCC patients divided by the recurrence status after hepatetomy.

Variable	ER group (n=54)	LR group (n=52)	NR group (n=79)
Gender (male/ female)	42/ 12	41/ 11	59/ 20
Age (years) ##	68.4 ± 1.4	67.4 ± 1.5	68.5 ± 1.2
HBV/ HCV/ NBNC(NASH/NAFLD)	5/ 11/ 40(36)	10/ 19/ 24(21)	14/ 36/ 31(26)
Child-Pugh score A/ B/ C	51/ 3/ 0	51/ 1/ 0	75/ 4/ 0
pStage I/ II/ III/ IV	2/ 15/ 21/16	11/ 24/ 13/ 4	12/ 52/ 12/ 3
Differentiation (well/ moderate/ poor)	2/ 32/ 20	5/ 35/ 12	9/ 52/ 18
Tumor size (cm) ##	7.5 ± 0.5	5.5 ± 0.5	3.8 ± 0.4
Vascular invasion (vp, vv, va) (yes/ no)	29/ 25	18/ 34	22/ 57
AFP (ng/ml) #	36.3(4.9-461)	11.4(5.2-117)	6.7(3.1-48.9)
DCP (mAU/ml) #	937(28.5-7461)	229(39-2286)	49(23-533)
Serum albumin (g/dl) ##	3.9 ± 0.1	4.0 ± 0.1	4.1 ± 0.1
Total bilirubin (mg/dl) ##	0.8 ± 0.1	0.8 ± 0.1	0.8 ± 0.1
ICG test (%)##	15.8 ± 1.4	15.0 ± 1.4	13.9 ± 1.1
Platelet ($\times 10^4/\mu l$) ##	19.3 ± 0.9	15.9 ± 0.9	16.1 ± 0.8
AST (IU/l) ##	45 ± 4	46 ± 5	43 ± 4
ALT (IU/l) ##	31 ± 4	42 ± 4	37 ± 3
Cr (mg/dl) ##	0.8 ± 0.1	0.8 ± 0.1	0.8 ± 0.1

ER, early recurrence; LR, late recurrence; NR, no recurrence; HBV, hepatitis B virus; HCV, hepatitis C virus; NBNC, non-B non-C hepatitis; NASH, non-alcoholic steatohepatitis; NAFLD, non-alcoholic fatty liver disease; pStage, pathologic stage; vp, portal vein invasion; vv, hepatic vein invasion; va, hepatic artery invasion; AFP, α-fetoprotein; DCP, des-γ-carboxy prothrombin; ICG, indocyanine green; AST, aspartate transaminase; ALT, alanine aminotransferase; Cr, creatinine.

#Data displayed as medians (25th to 75th percentile). ##All other values were expressed as means ± standard errors.

Supplementary Figure Legends

Supplementary Figure 1. Levels of MFG-E8 in liver tissues.

(A) Immunohistochemical analysis of cancerous and noncancerous liver tissues in hepatocellular carcinoma (HCC) patients. Scale bars, 100μm. (B,C) Comparision of milk fat globule-EGF factor 8 (MFG-E8) mRNA expression (n=50) (B) and MFG-E8 expression by western blotting (n=3) (C) in cancerous and non-cancerous liver tissues after surgical resection. Full-length blots are presented in Supplementary Fig.2. (D,E) Correlation between serum MFG-E8 levels and the MFG-E8 mRNA expression in cancerous and noncancerous liver tissues. ***p<0.001, Paired Student's t test was used for the analysis presented in Supplementary Fig. 1B.

GAPDH, glyceraldehyde-3-phosphate dehydrogenase.

Supplementary Figure 2. Full scans of western blots.

HV, healthy volunteer; HCC, hepatocellular carcinoma.

Supplementary Figure 3. The Minimal Information for Studies of Extracellular Vesicles 2018 (MISEV2018) checklist.

Supplementary Figure 1

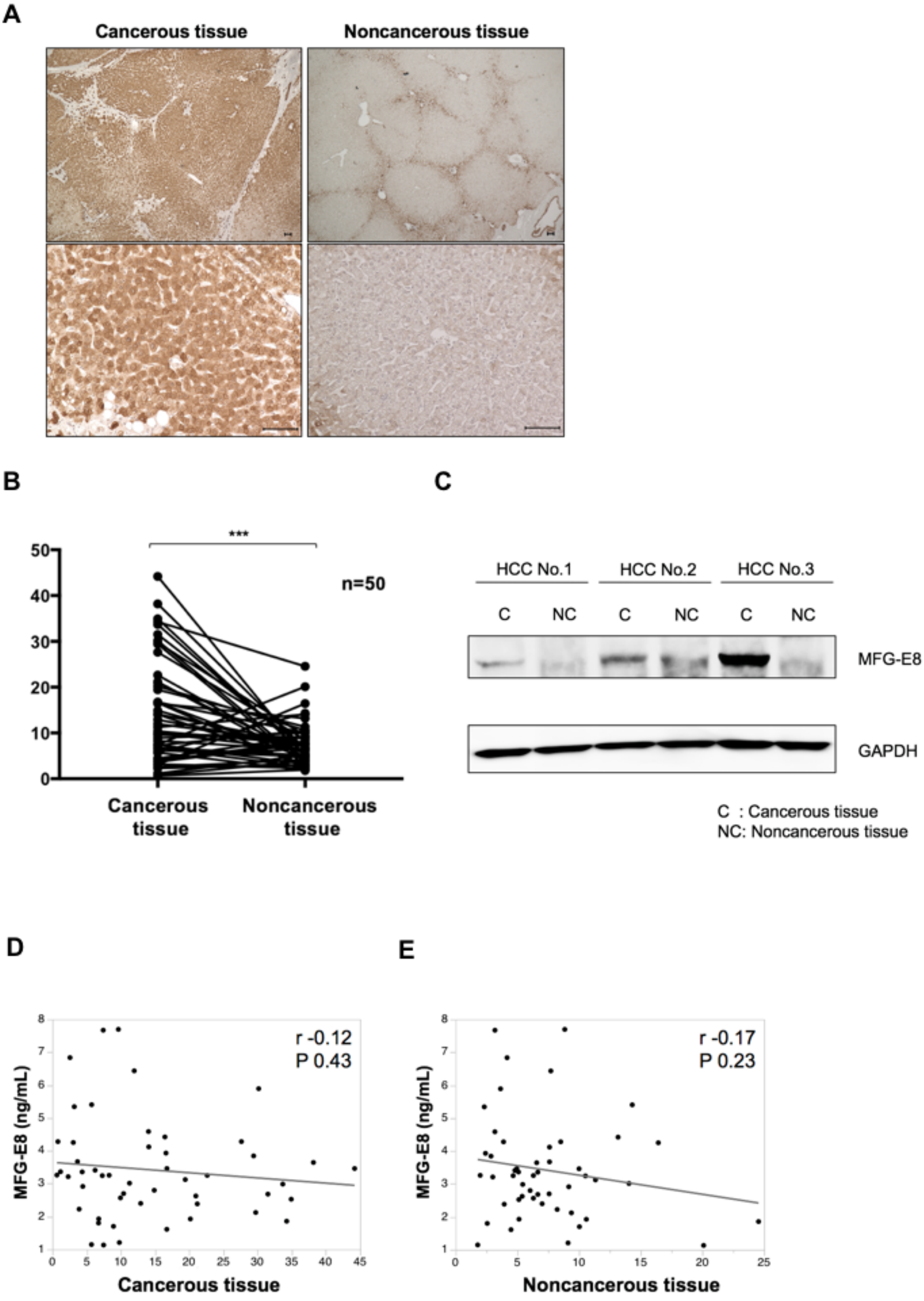

Supplementary Figure 2

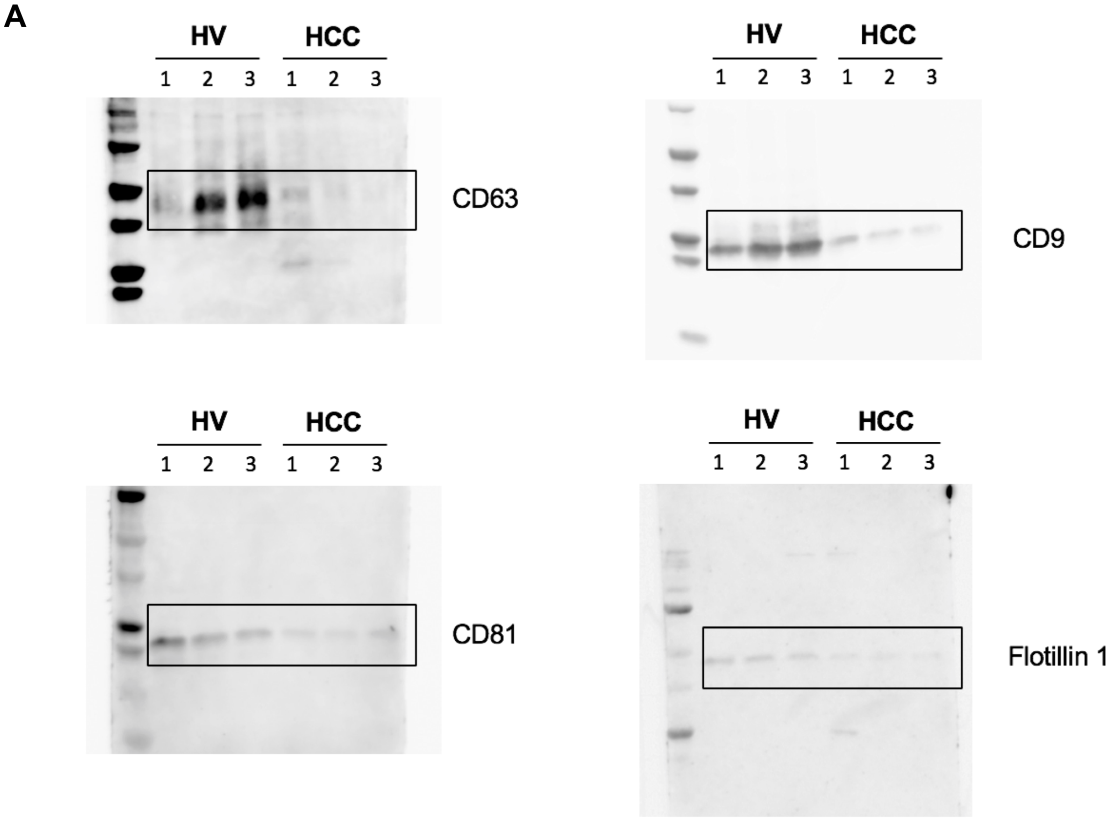

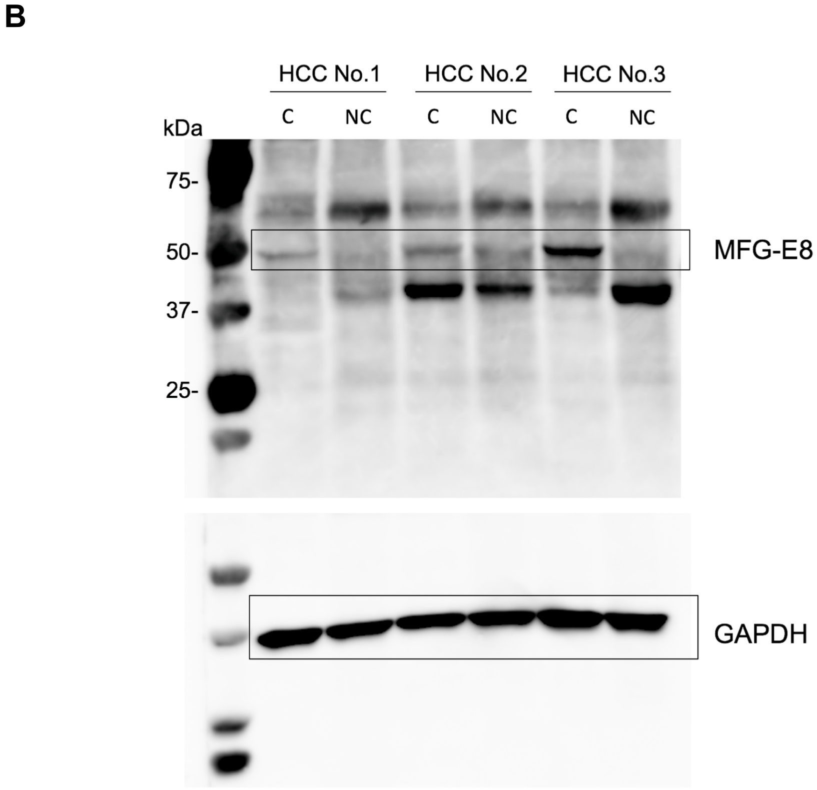

Supplementary Figure 3

MISEV2018 Checklist

Numbers refer to sections listed in the Table of contents from: C. Théry and K.W.Witwer, et al, ”Minimal Information for Studies of Extracellular Vesicles 2018 (MISEV2018): a posi- tion statement of the International Society for Extracellular Vesicles and update of the MISEV2014 guidelines”, J Extracell Vesicles 2018;7:1535750.

O Mandatory O Mandatory if applicable O Encouraged

1-Nomenclature

Mandatory

✓ O Generic term extracellular vesicle (EV): With demon- stration of extracellular (no intact cells) and vesicular nature per these characterization (Section 4) and function (Section 5) guidelines OR

O Generic term, e.g., extracellular particle (EP): no intact cells but MISEV guidelines not satisfied

Encouraged (choose one)

× O Generic term extracellular vesicle (EV) + specification (size, density, other)

× O Specific term for subcellular origin: e.g., ectosome, microparticle, microvesicle (from plasma membrane), exosome (from endosomes), with demonstration of the subcellular origin

× O Other specific term: with definition of specific criteria

2-Collection and pre-processing

Tissue Culture Conditioned medium (CCM, Section 2-a) O

n/a General cell characterization (identity, passage, myco- plasma check…)

n/a O Medium used before and during collection (additives, serum, other)

n/a O exact protocol for depletion of EVs/EPs from addi- tives in collection medium

n/a O Nature and size of culture vessels, and volume of medium during conditioning

n/a O specific culture conditions (treatment, % O2, coating, polarization…) before and during collection

n/a O Number of cells/ml or /surface area and % of live/ dead cells at time of collection (or at time of seeding with estimation at time of collection)

n/a O Frequency and interval of CM harvest

Biofluids or Tissues (Sections 2-b and -c)

✓ O Donor status if available (age, sex, food/water intake, collection time, disease, medication, other)

✓ O Volume of biofluid or volume/mass of tissue sample collected per donor

n/a O Total volume/mass used for EV isolation (if pooled from several donors)

✓ O All known collection conditions, including additives, at time of collection

✓ O Pre-treatment to separate major fluid-specific con- taminants before EV isolation

✓ O Temperature and time of biofluid/tissue handling before and during pre-treatment

n/a O For cultured tissue explants: volume, nature of med- ium and time of culture before collecting conditioned medium

n/a O For direct tissue EV extraction: treatment of tissue to release vesicles without disrupting cells

Storage and recovery (Section 2-d)

✓ O Storage and recovery (e.g., thawing) of CCM, bio- fluid, or tissue before EV isolation (storage tempera- ture, vessel, time; method of thawing or other sample preparation)

✓ O Storage and recovery of EVs after isolation (tempera- ture, vessel, time, additive(s)…)

3-EV separation and concentration

Experimental details of the method

✓ O Centrifugation: reference number of tube(s), rotor(s), adjusted k factor(s) of each centrifugation step (= time+ speed+ rotor, volume/density of centrifugation condi- tions), temperature, brake settings

n/a O Density gradient: nature of matrix, method of gen- erating gradient, reference (and size) of tubes, bottom- up (sample at bottom, high density) or top-bottom (sample on top, low density), centrifugation speed and time (with brake specified), method and volume of fraction recovery

n/a O Chromatography: matrix (nature, pore size,…), loaded sample volume, fraction volume, number

n/a O Precipitation: reference of polymer, ratio vol/vol or weight/vol polymer/fluid, time/temperature of incuba- tion, time/speed/temperature of centrifugation

✓ O Filtration: reference of filter type (=nature of mem- brane, pore size…), time and speed of centrifugation, volume before/after (in case of concentration)

✓ O Antibody-based : reference of antibodies, mass Ab/ amount of EVs, nature of Ab carrier (bead, surface) and amount of Ab/carrier surface

✓ O Other…: all necessary details to allow replication

n/a O Additional step(s) to concentrate, if any

n/a O Additional step(s) to wash matrix and/or sample, if any

Specify category of the chosen EV separation/concentra- tion method (Table 1):

O High recovery, low specificity = mixed EVs and non- EV components OR

O Intermediate recovery, intermediate specificity = mixed EVs with limited non-EV components OR

✓ O Low recovery, high specificity = subtype(s) of EVs with as little non-EV as possible OR

O High recovery, high specificity = subtype(s) of EVs with as little non-EV as possible

4-EV characterization

Quantification (Table 2a, Section 4-a)

✓ O Volume of fluid, and/or cell number, and/or tissue mass used to isolate EVs

✓ O Global quantification by at least 2 methods: protein amount, particle number, lipid amount, expressed per volume of initial fluid or number of producing cells/ mass of tissue

✓ O Ratio of the 2 quantification figures

Global characterization (Section 4-b, Table 3)

✓ O Transmembrane or GPI-anchored protein localized in cells at plasma membrane or endosomes

✓ O Cytosolic protein with membrane-binding or - association capacity

× O Assessment of presence/absence of expected contaminants (At least one each of the three categories above)

× O Presence of proteins associated with compartments other than plasma membrane or endosomes

× O Presence of soluble secreted proteins and their likely transmembrane ligands

× O Topology of the relevant functional components (Section 4-d)

Single EV characterization (Section 4-c)

✓ O Images of single EVs by wide-field and close-up: e.g. electron microscopy, scanning probe microscopy, super-resolution fluorescence microscopy

✓ O Non-image-based method analysing large numbers of single EVs: NTA, TRPS, FCS, high-resolution flow cytometry, multi-angle light-scattering, Raman spectroscopy, etc.

5-Functional studies

n/a O Dose-response assessment

n/a O Negative control = nonconditioned medium, biofluid/tissue from control donors, as applicable

n/a O Quantitative comparison of functional activity of total fluid, vs EV-depleted fluid, vs EVs (after high recovery/low specificity separation)

n/a O Quantitative comparison of functional activity of EVs vs other EPs/fractions after low recovery/high specificity separation

n/a O Quantitative comparison of activity of EV subtypes (if subtype-specific function claimed)

n/a O Extent of functional activity in the absence of contact between EV donor and EV recipient

6-Reporting

× O Submission of methodologic details to EV-TRACK (evtrack.org) with EV-TRACK number provided (strongly encouraged)

n/a O Submission of data (proteomic, sequencing, other) to relevant public, curated databases or open-access repositories

n/a O Data submission to EV-specific databases (e.g., EVpedia, Vesiclepedia, exRNA atlas)

× O Temper EV-specific claims when MISEV requirements cannot be entirely satisfied (Section 6-b)
